# Tin Carboxylate Complexes of Natural Bacteriochlorin for Combined Photodynamic and Chemotherapy of Cancer è

**DOI:** 10.3390/ijms222413563

**Published:** 2021-12-17

**Authors:** Sergey Tikhonov, Petr Ostroverkhov, Nikita Suvorov, Andrey Mironov, Yulia Efimova, Anna Plutinskaya, Andrei Pankratov, Anastasia Ignatova, Alexey Feofanov, Ekaterina Diachkova, Yuriy Vasil’ev, Mikhail Grin

**Affiliations:** 1 Department of Chemistry and Technology of Biologically Active Compounds, Medicinal and Organic Chemistry, Institute of Fine Chemical Technologies, MIREA-Russian Technological University, 86 Vernadsky Avenue, 119571 Moscow, Russia; mrp_ost@mail.ru (P.O.); suvorov.nv@gmail.com (N.S.); mironov@mitht.ru (A.M.); efimova@mirea.ru (Y.E.); michael_grin@mail.ru (M.G.); 2 P. Hertsen Moscow Oncology Research Institute—Branch of the National Medical Research Radiological Centre of the Ministry of Health of the Russian Federation, 2nd Botkinsky pr. 3, 125284 Moscow, Russia; anna2031@rambler.ru (A.P.); andreimnioi@yandex.ru (A.P.); 3 Laboratory of Optical Microscopy and Spectroscopy of Biomolecules of Shemyakin-Ovchinnikov Institute of Bioorganic Chemistry, Russian Academy of Sciences, Mikluko-Maklaya St. 16/10, 117997 Moscow, Russia; aignatova_83@mail.ru (A.I.); avfeofanov@yandex.ru (A.F.); 4 Biology Faculty, Lomonosov Moscow State University, Lenin Hills 1/12, 119234 Moscow, Russia; 5 Department of Oral Surgery of Borovsky Institute of Dentistry, I.M. Sechenov First Moscow State Medical University (Sechenov University), Trubetskaya St. Building 8\2, 119991 Moscow, Russia; 6 Department of Fundamental Medical Disciplines, Medical Faculty, Moscow Region State University (MRSU), St. Radio 10 Building 1, 105005 Moscow, Russia; 7 Department of Operative Surgery and Topographic Anatomy, I.M. Sechenov First Moscow State Medical University (Sechenov University), Trubetskaya St. Building 8\2, 119991 Moscow, Russia; y_vasiliev@list.ru; 8 Department of Prosthetic Dentistry, Dental Faculty, Kazan State Medical University of the Ministry of Health of Russia, St. Butlerova 49, 420012 Kazan, Republic of Tatarstan, Russia

**Keywords:** PDT, bacteriochlorin, chemotherapy, cancer, tin carboxylates

## Abstract

Photodynamic therapy (PDT) is currently one of the most promising methods of cancer treatment. However, this method has some limitations, including a small depth of penetration into biological tissues, the low selectivity of accumulation, and hypoxia of the tumor tissues. These disadvantages can be overcome by combining PDT with other methods of treatment, such as radiation therapy, neutron capture therapy, chemotherapy, etc. In this work, potential drugs were obtained for the first time, the molecules of which contain both photodynamic and chemotherapeutic pharmacophores. A derivative of natural bacteriochlorophyll a with a tin IV complex, which has chemotherapeutic activity, acts as an agent for PDT. This work presents an original method for obtaining agents of combined action, the structure of which is confirmed by various physicochemical methods of analysis. The method of molecular modeling was used to investigate the binding of the proposed drugs to DNA. In vitro biological tests were carried out on several lines of tumor cells: Hela, A549, S37, MCF7, and PC-3. It was shown that the proposed conjugates of binary action for some cell lines had a dark cytotoxicity that was significantly higher (8–10 times) than the corresponding metal complexes of amino acids, which was explained by the targeted chemotherapeutic action of the tin (IV) complex due to chlorin. The greatest increase in efficiency relative to the initial dipropoxy-BPI was found for the conjugate with lysine as a chelator of the tin cation relative to cell lines, with the following results: S-37 increased 3-fold, MCF-7 3-fold, and Hela 2.4-fold. The intracellular distribution of the obtained agents was also studied by confocal microscopy and showed a diffuse granular distribution with predominant accumulation in the near nuclear region.

## 1. Introduction

The limitations of existing methods for the treatment of oncological diseases make it necessary to search for new ways of treating tumors, including combinations of methods well known in oncology [1]. Chemotherapy is one of the popular methods for treating cancer tumors of various nosologies and localizations [2]. Cytostatics damage the rapidly proliferating tumor cells. However, the cells of some healthy tissues have a physiologically determined high frequency of division and are damaged by chemotherapy; as a result, side effects are observed. This primarily affects short-lived granulocytes (neutropenia), then platelets (thrombocytopenia), and, ultimately, erythrocytes (anemia). Infertility is caused by the inhibition of spermatogenesis or egg maturation. The majority of cytostatics affect the DNA metabolism; hence, there is a danger of damaging the genetic material of healthy cells, i.e., mutagenic action. If cytostatics are prescribed during pregnancy, fetal development is impaired (teratogenic effect) [3]. All the above side effects of chemotherapy can be avoided if the targeting effect of anticancer drugs is implemented [4]. It was discovered at the beginning of the twentieth century that cancer cells could selectively retain and accumulate macroheterocyclic compounds such as porphyrins, and it was found later that hydrogenated analogues of porphyrins—chlorins and bacteriochlorins—had a tumorotropic effect [5,6,7,8,9,10]. Moreover, the abovementioned pigments are capable of generating singlet oxygen and other reactive radicals that have a toxic effect on tumor cells upon local exposure to laser irradiation at a certain wavelength [11]. The efficiency of photodynamic damage to a sensitized cell is determined by the intracellular concentration of the sensitizer, its localization in the cell, photochemical activity, and the applied dose of laser irradiation [12]. In addition to the direct cytotoxic effect on tumor cells, an important role in tumor destruction in photodynamic therapy is occupied by the violation of blood supply due to damage to the endothelium of blood vessels of the tumor tissue, cytokine reactions caused by stimulation of the production of the tumor necrosis factor, the activation of macrophages, leukocytes, and lymphocytes [13,14]. The high selectivity of tumor damage upon PDT allows the traumatizing of the surrounding healthy tissues to be minimized, which is demonstrated by high functional and cosmetic results of treatment [15,16].

The main requirement for PSs used as agents for PDT is the absorption of the latter in the red and near-IR spectral regions. Chlorins and bacteriochlorins, absorbing in the region of 650–850 nm, ensure the penetration of light into the depth of the tissue up to 2 cm, which realizes the treatment of deep-lying and pigmented tumors. Modification of the macrocycle periphery can change the conjugation system and lead to a bathochromic shift of the long-wavelength absorption band. In addition, the ability of the abovementioned PSs to fluoresce when irradiated in the absorption band makes them ideal theranostics for use in oncology [17,18].

The idea of combined photodynamic and chemotherapy for the treatment of solid tumors has been successfully implemented previously [19,20,21]. For example, the combined treatment of PDT and cisplatin has been shown to be more efficient than either of the monotherapies alone. It is also important that the suggested combined treatment features a synergistic effect and, moreover, minimizes the damaging effect of cisplatin by reducing its dose without sacrificing the treatment efficiency [22].

Yet another approach to increasing the efficiency of anticancer therapy involves combining the photodynamic subunit represented by porphyrin or its hydrogenated analogs with a chemotherapeutic agent, for example, complexes of gold, tin, platinum, etc., in a single drug. As shown in studies on porphyrin–cisplatin conjugates, the latter exhibit a synergistic effect [20]. Tests on cell lines of bladder cancer TCC-SUP/J82 and breast cancer MDA-MB-231 showed that the conjugates had a higher photodynamic efficiency than cisplatin.

The advantage of combined therapy in comparison with monotherapy lies in the targeted delivery of chemotherapy drugs to the tumor location due to binding with porphyrin, and hence, a decrease in the therapeutic dose of the drug and a decrease in systemic toxicity to the body. Yet another problem of chemotherapy involves the multidrug resistance of tumor cells [23]. Resistance develops due to various reasons, which can include weakening the drug capture by the cell; increasing the protective transport from the cell, e.g., due to an increase in P-glycoprotein production; weakening the bioactivation of the drug required for its transition to the active form; and increasing the efficiency of DNA repair mechanisms upon damage enacted by cisplatin [24]. In such case, PDT destroys the resistant tumor cells that survived the chemotherapy.

The use of amino acids in the design of antitumor pharmaceutical compounds is caused by the enhanced selectivity of their accumulation in a tumor. For example, mono *L*-aspartylchlorin e_6_ (Talaporfin) is a second-generation PS, and owing to addition of aspartic acid, it is superior to unmodified chlorin e6 in pharmacodynamic parameters, inhibition of tumor growth and increasing the lifespan of tumor-bearing animals [25]. In addition, the structure of amino acids makes them potential complexing agents for the cations of various metals and organometallic complexes that possess antitumor activity themselves [26].

Lately, organotin compounds have been considered as promising antineoplastic agents and an alternative to platinum-containing cytostatics [27]. Cell membranes (including mitochondrial ones), as well as tubulin protein and DNA, are the most thoroughly studied targets for organotin compounds [28]. Organotin carboxylates are stable at physiological pH values and are not demetallized in the blood stream while exhibiting high antiproliferative activity against tumor cells of various genesis, including platinum-resistant tumors. At the same time, their toxicity is significantly lower than that of platinum-based formulations [29].

Amino-acid–tin carboxylate complexes can act as independent pharmaceutical agents but can also be included in more complex structures. The diversity of amino acids, including non-proteinogenic ones, and their metal complexes makes it possible to obtain pharmaceutical agents for various cellular targets. This is a promising approach in the combat against tumors of various etiologies [30].

Previously, our scientific team obtained amino acid derivatives of natural pigments [31]. In a continuation of this approach, now we report a synthesis and study of the properties of bacteriochlorophyll *a* derivatives with tin carboxylate complexes.

## 2. Results and Discussions

### 2.1. Chemistry

Two amino acids were chosen as ligands for the synthesis of tin complexes: non-proteinogenic *p*-aminobenzoic acid and *L*-lysine protected at the α- and ε-amino groups.

**Scheme 1:**
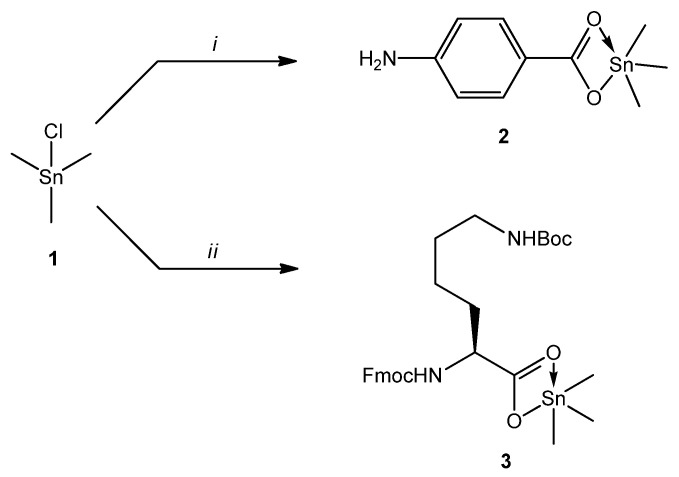
Reagents and conditions: *i*: *p-*Aminobenzoic acid, CH_3_OH, 1M KOH, 45 °C, 96 h; *ii: N*-α-*Fmoc-N*-ε-*Boc*-Lysine, CH_3_OH, 1 M KOH, 45 °C, 96 h.

In the first stage of our work, tin complexes with the abovementioned amino acids were obtained using well-known methods (Scheme 1) [32]. It was found experimentally that the time required to complete the reaction of amino acids with trimethyltin chloride and reach the maximum product yield amounted to 96 h. Even though metal complexes based on *para-*aminobenzoic acid are well studied [33], we are the first to obtain their conjugates with a derivative of natural bacteriochlorophyll *a*.

Characteristic bands of stretching vibrations of O-Sn and C-Sn bonds, as well as the bands of vibrations of C-O bonds characteristic of carboxylate groups, were found in the IR spectra of complexes **2** and **3**.

The proton signals of three methyl groups bound to the tin atom were detected in the ^1^H NMR spectra at 0.45 ppm for complex **2** and at 0.38 ppm for complex **3**. In the mass spectra of complexes **2** and **3**, signals of their molecular ions with isotopic splitting characteristic of the tin cation were observed.

**Scheme 2:**
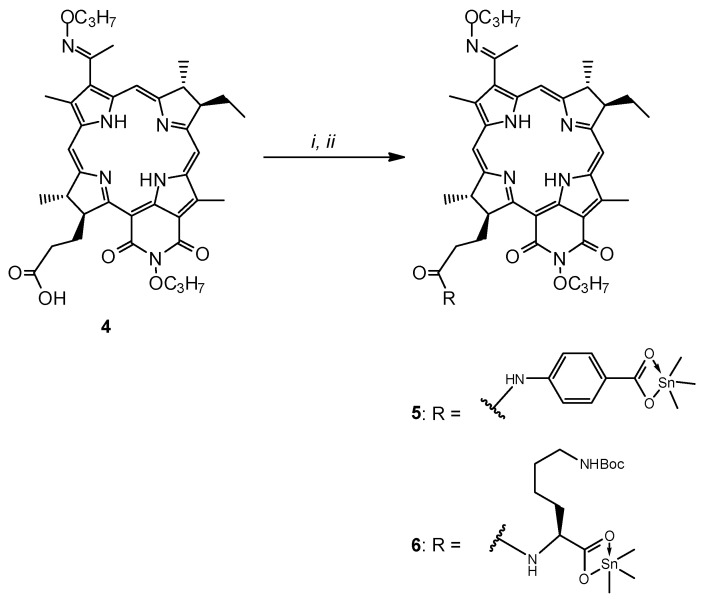
Reagents and conditions: *i:* CH_2_Cl_2_, EDC, NHS, 0 °C, 30 min, compound **2**, Et_3_N, 48 h; *ii*: CH_2_Cl_2_, EDC, NHS, 0 °C, 30 min, compound **3**, Et_3_N, 48 h.

At the second stage of this work, we obtained conjugates of dipropoxybacteriopurpurinimide (dipropoxy-BPI) **4**, a leader compound that showed high photodynamic efficiency in in vitro and in vivo studies [34], with amino acid complexes of tin (IV), whose synthesis is described above. The carbodiimide synthesis method was used to create an amide bond between the amino group of *p*-aminobenzoic acid or the α-amino group of lysine on the one hand, and the carboxy group of the propionic residue at position 17 of the macrocycle on the other hand. In the case of the complex with lysine, *Fmoc* protection was first selectively removed from the α-amino group under alkaline conditions, while retaining *Boc* protection at the ε-amino group of lysine (Scheme 2).

The resulting products **5** and **6** were isolated by preparative TLC. Their structures were confirmed by a set of physicochemical methods of analysis.

**Figure 1:**
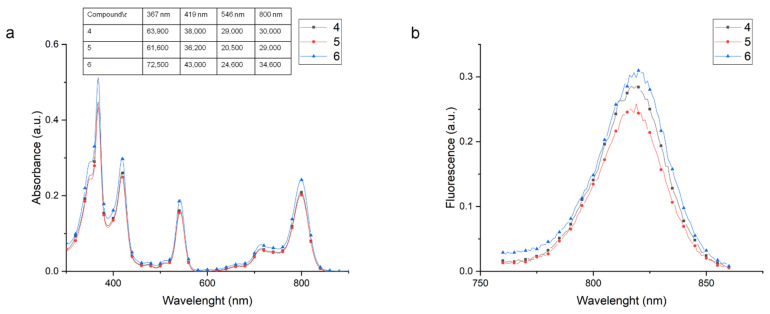
Electronic absorption (**a**) and fluorescence (**b**) spectra of the compounds obtained.

A study of photophysical properties showed that in the case of the conjugate of dipropoxy–BPI with the Sn complex of *p*-aminobenzoic acid **5**, a new maximum (λ = 267 nm) corresponding to the absorption of the benzene ring appeared in the electronic absorption spectra, whereas the pigment with the Sn–lysine complex **6** showed no changes in the absorption bands (Figure 1a).

The fluorescence spectrum of dipropoxy–BPI with tin complexes did not differ from that of the starting pigment (Figure 1b).

### 2.2. Docking

Molecular docking is an important tool in drug development. It plays a significant role in understanding the mechanism of the interaction between the ligand being studied and the corresponding biological target that can be represented by a specific DNA sequence or by specific protein binding sites. It should be noted that DNA is not a typical target for PS; however, in the case of photosensitizers modified with various DNA-targeted ligands, the interaction of such molecules with DNA was identified [35].

To understand the mechanism of interaction of the complexes with DNA obtained, they were docked with the sequence d(CGCGAATTCGCG)2 of a DNA dodecamer duplex (PDB ID: 1BNA). The results of the docking tests showed that the complexes studied interacted with B-DNA (PDB ID: 1BNA) mainly in the minor groove region and were stabilized due to van der Waals forces and hydrophobic interactions with functional DNA groups.

**Figure 2:**
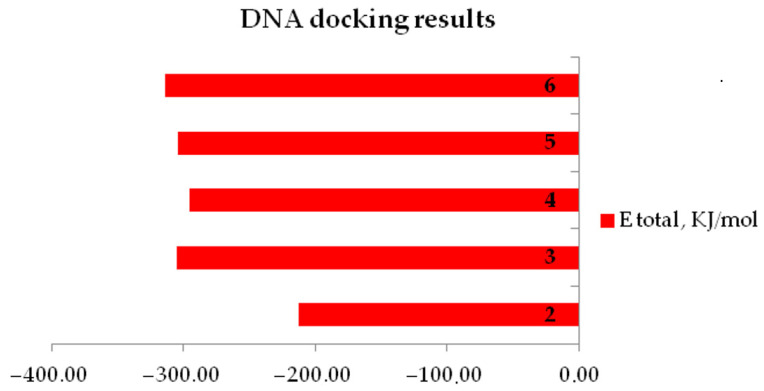
Results of docking compounds **2**–**6** with DNA.

The results of calculations on the energies of ligand-targeted bonds are presented in the summary plot below (Figure 2).

It follows from the results of molecular docking that all the compounds obtained in this work have a high energy of binding with the DNA molecule.

### 2.3. DNA Binding Test

To estimate the interaction between single-stranded DNA (SS-DNA) and compounds **2**, **3**, **4**, **5**, and **6** obtained in this work, we used absorption spectroscopy in the UV-visible spectrum region [36]. If a compound is intercalated into DNA, a shift of absorption bands to the long-wavelength (red) region occurs (which usually depends on the strength of intercalation interaction) accompanied by a decrease in absorption intensity due to a strong stacking interaction between the nitrogen bases of DNA and the aromatic chromophore of the compound [37].

Based on optical density measurements, the internal binding constants of the compounds to DNA were determined using the Benesi–Hildebrand equation:

(1) $$\frac{A_{0}}{A-A_{0}}=\frac{\varepsilon_{G}}{\varepsilon_{H-G}-\varepsilon_{G}}+\frac{\varepsilon_{G}}{\varepsilon_{H-G}-\varepsilon_{G}}\times \frac{1}{K\left[DNA\right]}$$

where *K* is the association/binding constant, *A_0_* and *A* are the absorption of the compound and its complex with DNA, respectively, while *ε_G_* and *ε_H-G_* are the absorption coefficients of the compound and the compound–DNA complex, respectively. The free Gibbs energy was determined from the equation:

(2) ΔG=−RT lnK

where *R* is the total gas constant (8.314 J·K^−1^·mol^−1^) and *T* is the temperature (298 K).

It follows from the results presented in Table 1 that the conjugate of dipropoxy–BPI with the Sn complex of lysine 6 is the leading compound in terms of the efficiency of binding to DNA.

### 2.4. Biology

To determine the biological activity of the obtained compounds **2**–**6**, we performed in vitro studies on the following tumor cell lines: prostate gland adenocarcinoma (PC-3); breast adenocarcinoma (MCF-7); lung carcinoma (A549); cervix adenocarcinoma (Hela); and mouse sarcoma (S-37). Both dark toxicity and photoinduced cytotoxicity were studied.

One can see from the data presented in Table 2 that the dark cytotoxicity of conjugates **5** and **6** in some cell lines is considerably higher than that of the corresponding complexes. Apparently, the addition of a chemotherapeutic agent to a transporter such as bacteriochlorin favors better internalization and accumulation of conjugates in tumor cells and, hence, boosts the cytotoxicity.

The photoinduced cytotoxicity study showed that conjugates were most efficient upon irradiation followed by incubation with cells for 48 h. The largest increase in efficiency compared to the initial dipropoxy–BPI **4** was found for conjugate **6** for the cell lines, with the following results: 3-fold for S-37, 3-fold for MCF-7, and 2.4-fold for HeLa (Table 3). At the same time, no such increase in photoinduced cytotoxicity was observed for conjugate **5** of dipropoxy–BPI with the Sn complex of *p-*aminobenzoic acid. The results of biological studies correlate well with the results of molecular docking, according to which the maximum efficiency was predicted for PS **6**.

**Figure 3:**
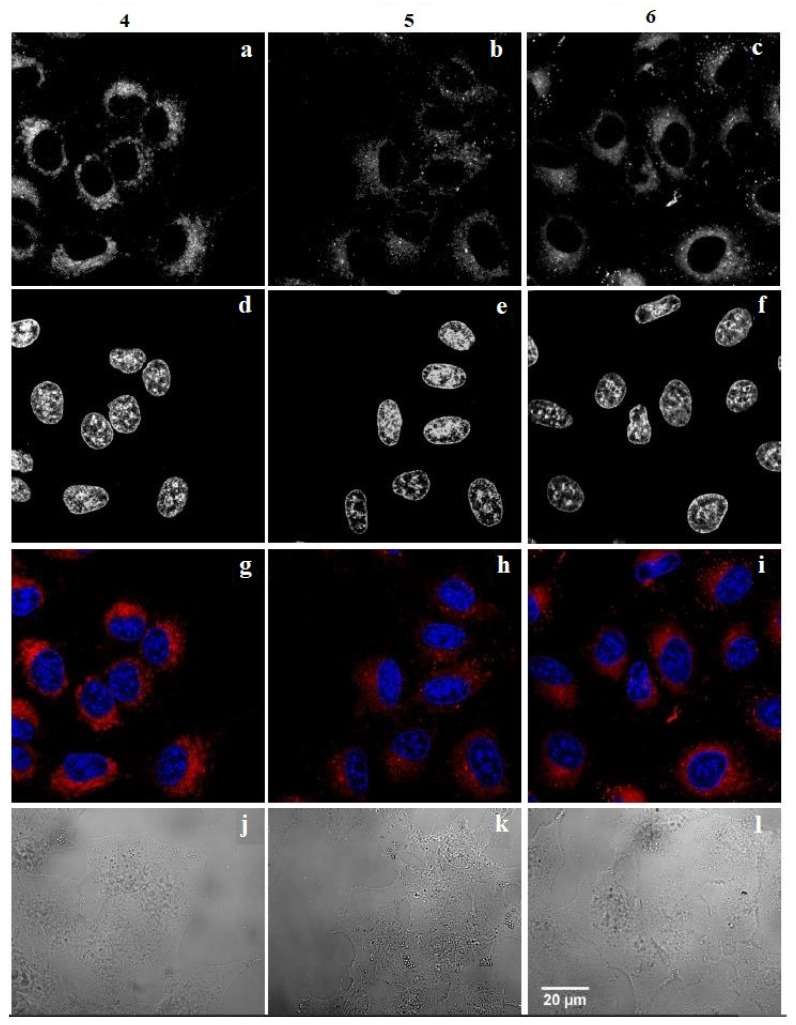
Distribution of **4**, **5,** and **6** in A549 cells: (**a**–**c**) fluorescent images of **4**, **5,** and 6; (**d**–**f**) fluorescent images of Hoechst 33,342 stain; (**g**–**i**) overlay of fluorescent images (blue—Hoechst 33,342; red—**4**, **5,** or **6**); (**j**–**l**) cell images in transmitted light.

Studies of the intracellular distribution of compounds **4**, **5**, and **6** were performed with confocal laser scanning fluorescence microscopy. The corresponding fluorescent images of A549 cells are shown in Figure 3. It was found that the compounds were distributed in the cytoplasm of A549 cells (Figure 3a–c) in a diffuse granular manner with predominant accumulation in the perinuclear region. No co-localization of **4**, **5**, or **6** with the Hoechst 33342 nuclear stain was found. Fluorescence of the conjugates in cell nuclei was absent or as beyond the instrument sensitivity.

## 3. Materials and Methods

### 3.1. Chemistry

The solvents were purified and prepared using standard procedures. Trimethyltin chloride was purchased from Acros Organic (Moscow, Russia). *O*-Propyloxime-*N*-propoxybacteriopurpurinimide was obtained using the standard method developed in our laboratory [34]. TLC was performed using 60 Merck silica gel (Darmstadt, Germany) on 20 × 20 cm^2^ plates with a thickness of 1 mm. Mass spectra were recorded on a Bruker Ultraflex TOF/TOF mass spectrometer (Bremen, Germany) with the MALDI method using 2,5-dihydroxybenzoic acid (DHB) as a matrix, as well as on a TSQ QuantumAccess MAX triple quadrupole mass spectrometer (Waltham, MA, USA) by the ESI method. NMR spectra were recorded on a Bruker DPX-300 spectrometer (Bremen, Germany) at 25 °C in DMSO-d6 at a working frequency of 300 MHz. Residual signals of ^1^H nuclei were used to calibrate the scale. Experiments were performed according to standard Bruker methods. IR spectra were obtained on an Infralum FT-08 FT-IR spectrometer (Saint Petersburg, Russia) in KBr pellets. Fluorescence spectra were obtained on a Lumex Fluorat-02 PANORAMA spectrofluorimeter (Saint Petersburg, Russia) in dichloromethane at an excitation wavelength of 530 nm. Electronic absorption spectra were obtained from ethanol on an SF-2000 spectrophotometer manufactured by OKB SPEKTR (Saint Petersburg, Russia).

#### 3.1.1. Synthesis of Tin Complex Based on 4-Aminobenzoic Acid (2)

4-Aminobenzoic acid (137 mg, 1 mmol) was dissolved in methanol (3 mL) and 1 M KOH (1 mL). Trimethyltin chloride (200 mg, 1 mmol) was added to the resulting solution. The reaction was performed for 96 h at 40 °C with stirring. The progress of the reaction was monitored by TLC. After cooling the reaction mixture, the solvent was removed in vacuo. A small amount of chloroform was added to the solid residue. The resulting suspension was filtered off. The filtrate was concentrated in vacuo. The product was recrystallized from a petroleum ether–ethanol mixture (1/1, *v*/*v*). The yield of the target compound (**2**) was 78 mg (57%). MALDI MS *m*/*z* (M + Na^+^): calculated for C_10_H_15_NO_2_SnNa^+^ 324.00, found 323.73, (M + K^+^): calculated for C_10_H_15_NO_2_SnK^+^ 339.98, found 339.72; IR: ν (O-Sn) 429 cm^−1^, ν (C-Sn) 505 cm^−1^, ν (COO^-^ sym.) 1516 cm^−1^, ν (COO^-^ asym.) 1647 cm^−1^; ^1^H NMR (300 MHz, DMSO-d6) δ 7.57 (d, 2H, *J* = 7.7 Hz), 6.49 (d, 2H, *J* = 7.7 Hz), 5.57 (br. s, 2H), 0.45 (t, 9H, *J* = 34.2 Hz).

#### 3.1.2. Synthesis of Tin Complex Based on Lysine (3)

α-Fmoc-ε-Boc-Lys (234 mg, 0.5 mmol) was dissolved in methanol (3 mL) and 1 M KOH (0.5 mL). Trimethyltin chloride (100 mg, 0.5 mmol) was added to the solution. The reaction was performed for 96 h at 40 °C with stirring. The progress of the reaction was monitored by TLC. After cooling the reaction mixture, the solvent was removed in vacuo. A small amount of chloroform was added to the solid residue. The resulting suspension was filtered off. The filtrate was concentrated in vacuo. The product was recrystallized from a petroleum ether–ethanol solvent mixture (1/1, *v*/*v*). The yield of the target compound (**3**) was 115 mg (49%). MALDI MS *m*/*z* [M + K^+^]: calculated for C_29_H_40_N_2_O_6_SnK^+^671.15, found 670.89; IR: ν(O-Sn) 548 cm^−1^, ν(C-Sn) 621 cm^−1^, ν(COO^−^ sym.) 1248 cm^−1^, ν (COO^−^ asym.) 1516 cm^−1^; ^1^H NMR (300 MHz, DMSO-d6) δ 7.13–7.91 (m, 8H), 6.73 (m, 1H), 5.73 (m, 1H), 4.20 (m, 2H), 3.76 (m, 1H), 3.36 (m, 1H), 2.87 (q, 2H, *J* = 6.0 Hz), 1.15–1.42 (m, 15H), 0.38 (t, 9H, *J* = 34.2 Hz).

#### 3.1.3. Synthesis of the Conjugate of O-Propyloxime-*N*-Propoxybacteriopurpurinimide with the Tin Complex of 4-Aminobenzoic Acid (5)

*O-*Propyloxime-*N-*propoxybacteriopurpurinimide (35 mg, 0.05 mmol) was dissolved in dichloromethane (3 mL). 1-Ethyl-3-(3-dimethylaminopropyl)carbodiimide (14 mg, 0.075 mmol) and *N*-hydroxysuccinimide (15 mg, 0.13 mmol) were added to the resulting solution. After that, the reaction mixture was stirred at 0 °C for 1 h under argon. Next, complex **2** (90 mg, 0.3 mmol) and triethylamine (40 μL, 0.02 mmol) were added to the mixture. The reaction was performed for 48 h with continuous stirring. The target product was purified by column chromatography in a chloroform–methanol eluent system (30/1, *v*/*v*). The yield of the target compound (**5**) was 15 mg (43%). MALDI MS *m/z* [M + H^+^]: calculated for C_49_H_61_N_7_O_7_Sn 979.37, found 978.74; IR spectrum of the compound: ν (O-Sn) 504 cm^−1^, ν (C-Sn) 548 cm^−1^, ν (COO^−^ sym.) 1288 cm^−1^, ν (COO^−^ asym.) 1602 cm^−1^; ^1^H NMR (300 MHz, DMSO-d6) δ 8.62 (s, 1H), 8.55 (s, 1H), 8.38 (s, 1H), 7.90 (d, 2H, *J* = 7.6 Hz), 6.65 (d, 2H, *J* = 7.6 Hz), 5.23 (m, H), 4.46 (m, 4H), 4.24 (m, 1H), 4.19 (m, 1H), 4.12 (m, 1H), 4.02 (m, 1H), 3.64 (s, 3H), 3.29 (s, 2H), 2.75 (s, 2H), 2.42 (m, 2H), 2.34 (m, H), 2.00–2.09 (m, 6H), 1.80 (m, 3H), 1.69 (m, 3H), 1.14–1.26 (m, 9H), 0.58 (t, 9H, *J* = 34.19 Hz), 0.22 (br. s, 2H). UV/VIS (CH_2_Cl_2_) λ_max_, nm (ε, M^−1^ cm^−1^): 367 (61,600), 419 (36,200), 546 (20,500), 800 (29,000).

#### 3.1.4. Synthesis of the Conjugate of O-Propyloxime-*N*-Propoxybacteriopurpurinimide with the Tin Complex of Lysine (6)

*O-*Propyloxime-*N-*propoxybacteriopurpurinimide (35 mg, 0.05 mmol) was dissolved in dichloromethane (5 mL). 1-Ethyl-3-(3-dimethylaminopropyl)carbodiimide (14 mg, 0.075 mmol) and *N*-hydroxysuccinimide (15 mg, 0.13 mmol) were added to the resulting solution. After that, the reaction mixture was stirred at 0 °C for 1 h under argon. Next, complex **3** (90 mg, 0.3 mmol) and triethylamine (40 μL, 0.02 mmol) were added to the mixture. The reaction was performed for 48 h with continuous stirring. The target product was purified by column chromatography in a chloroform–methanol eluent system (30/1, *v*/*v*). The yield of the target compound (**6**) was 13 mg (37%).

MALDI MS *m*/*z* [M + H^+^]: calculated for C_53_H_76_N_8_O_9_Sn 1088.48, found 1088.53; IR: ν(O-Sn) 574 cm^−1^, ν(C-Sn) 675 cm^−1^, ν(COO^−^ symm.) 1115 cm^−1^, ν(COO^−^ asym.) 1546 cm^−1^; ^1^H NMR (300 MHz, DMSO-d6) δ 8.39 (s, 1H), 8.62 (s, 1H), 8.55 (s, 1H), 5.11 (m, 2H), 4.45 (m, 4H), 4.34 (m, 1H), 4.19 (m, 1H), 4.13 (m, 1H), 4.02 (m, 1H),3.75 (m, 1H), 3.69 (m, 2H), 3.64 (s, 3H), 3.52–3.61 (m, 6H), 3.29 (s, 2H), 2.75 (s, 2H), 2.34 (m, 2H), 1.88–2.09 (m, 6H), 1.81 (m, 3H), 1.68 (m, 3H), 1.09–1,26 (m, 9H), 0.00 (m, 18H), −0.07 (brs, 1H), −0.16 (brs, 1H). UV/VIS (CH_2_Cl_2_), λ_max_, nm (ε, M^−1^ cm^−1^): 367 (72,500), 419 (43,000), 546 (24,600), 800 (34,600).

### 3.2. Molecular Docking

Molecular docking studies were performed using HEX 8.0.0 software (Dave Ritchie, Paris, France). Receptor preparation was performed using UCSF Chimera 1.15 software (Resource for Biocomputing, Visualization, and Informatics University of California, San Francisco, CA, USA). The crystal structure of B-DNA (PDB ID: 1BNA) was obtained from the protein databank (http://www.rcsb.org./pdb, accessed on 20 October 2020). The parameters used for docking were as follows: ShapeOnly correlation type, OPLS Minimisation post-processing, FFT 3D mode, grid size 0.6, receptors range 180, ligands range 180, rotation range 360, and distance range 40.

### 3.3. DNA Binding Test

Single-stranded DNA (ss-DNA) was dissolved in deionized water by stirring for 12 h (pH = 7.0) and stored at 4 °C. The buffer solution was prepared using deionized water (20 mM of phosphate buffer NaH_2_PO_4_–Na_2_HPO_4_, pH = 7.2). To confirm that the DNA was largely protein-free, an absorbance value of 2.5115 at a wavelength of 261 nm was obtained for a solution of ss-DNA in the buffer. The DNA concentration was determined by the Bouguer–Lambert–Beer law, according to which at a molar absorption coefficient of 6600 M^−1^ cm^−1^ (261 nm), the DNA concentration is 7.6 × 10^−3^ M. The compounds were dissolved in 70% ethanol. The concentration of the compounds studied was 23.8 μM. Absorption measurements in the UV-visible region were performed at a fixed concentration of compounds while the concentration of the DNA solution was varied (1–40 µM). In order to exclude the absorption signals of the DNA itself in the measurement of control samples, reference samples containing the same volume of DNA of a given concentration were also used. Before an experiment, the solutions of the complexes were incubated with DNA for about 5 min at room temperature.

Absorption spectra were recorded in quartz cells (1 cm) at room temperature (25 ± 1 °C) at 1 nm steps over 3 scans.

### 3.4. Biology

#### 3.4.1. Photoinduced and Dark Cytotoxicity Studies

The following human cell cultures were used in the experiments: prostate adenocarcinoma (PC-3); breast adenocarcinoma (MCF-7); lung carcinoma (A-549); cervix adenocarcinoma (Hela); and mouse sarcoma (S-37). Tumor cells were incubated in plastic flasks with a cell growth surface of 25 cm^2^ (Costar, Corning, NY, USA) in DMEM (S-37 cell culture), RPMI-1640 (PC-3 cell culture), or Eagle media (MCF-7 and A-549) with L-glutamine supplemented with 10% fetal calf serum (FBS) (PanEco, Moscow, Russia). Incubation of cells was carried out at 37 °C in a humidified atmosphere containing 5% CO_2_ (Binder CO_2_ incubator, Tuttlingen, Germany). Cell lines from 3 to 7 passages were used. In experiments for the assessment of the photoinduced activity of compounds, cells were cultured into 96-well culture plates at a concentration of 10^5^ cells per ml (when incubated for 24 h after exposure to light) or 7 × 10^4^ cells per ml (when incubated for 48 h after irradiation). They were incubated for 28–30 h at 37 °C (humidified atmosphere, 5% carbon dioxide). Next, solutions of compounds were added to a final concentration from 0.03 μg/mL to 20 μg/mL. After 4 h of incubation with photosensitizers, cells were irradiated with a halogen lamp through a KS-19 broadband filter (λ ≥ 720 nm). The power density was 19.5 ± 1.0 mW/cm^2^, and the calculated light dose was 10 J/cm^2^. After irradiation was completed, the plates with the cells were placed in a CO_2_ incubator for 24 or 48 h. To estimate cytotoxic activity, cells were incubated with compounds under dark conditions for selected time periods. Cell survival was estimated using a colorimetric MTT test. Based on the results, IC_50_ values were calculated, i.e., the PS concentrations at which 50% cell death occurred after exposure. Quantitative parameters were calculated from the results of three independent tests.

#### 3.4.2. Study of Intracellular Distribution

A549 cells were incubated at 37 °C at 5% CO_2_ and 100% humidity in a DMEM/F12 medium (PanEco, Moscow, Russia) with addition of 2 mM of glutamine and 5% fetal bovine serum. Reseeding was performed two times a week. On the day before the experiment, the cells were cultured on cover glasses in the wells of a 24-well plate in an amount of 10^5^ cells/well.

Cells were incubated with 0.5 μM of compounds **4**, **5**, or **6** for 3 h. In the last 15 min of incubation, 5 μM of the Hoechst 33342 stain, which selectively stains cell nuclei, was added.

Measurements were carried out with a Zeiss LSM 710 confocal microscope (Oberkochen, Germany). A laser with a wavelength of 488 nm was used for fluorescence excitation of **4**, **5**, or **6**. Fluorescence was detected in the range of >650 nm using a highly sensitive APD detector. To excite the fluorescence of Hoechst 33,342 dye, a laser with a wavelength of 405 nm was used. Fluorescence was detected in the range of 415–500 nm.

## 4. Conclusions

In this work, conjugates of tin (IV) carboxylate complexes with a derivative of natural bacteriochlorophyll a, dipropoxy-BPI, were synthesized. The proposed synthesis scheme includes the initial creation of tin complexes with p-aminobenzoic acid and ε-Boc-protected lysine and their subsequent addition to bacteriopurpurinimide. An alternative synthesis route consisted of the preparation of amino acid derivatives of the BPI and their metallation with tin trimethyl chloride. However, the second route turned out to be unacceptable because of the harsh conditions of tin introduction to the periphery of the macrocycle and the high probability of the oxidation of the latter.

Taking DNA as a possible molecular target of the obtained complexes, molecular docking was implemented in this work, which showed the most efficient binding of metal complex 6 to nucleic acid, which was confirmed by the results of biological tests on five cell lines of tumors of various genesis. The study of dark and photoinduced cytotoxicity showed an additive effect on tumor cells of tin (IV) complexes with amino acid ligands and photodynamic action of bacteriopurpurinimide when irradiated in its absorption band at 800 nm.

Apparently, the BPI plays a double role—the implementation of targeted transport of the metal complex into cancer cells and their photoinduced destruction.

## Figures and Tables

**Table 1:** Results of docking and DNA binding test for compounds **2**–**6**.

Compounds	Docking	Binding Test	
	Total Interaction Energy, kJ·mol^−1^	Binding Constant, *K* × 10^6^ M^−1^	Gibbs Energy, kJ·mol^−1^
**2**	−212.20	1	−41.3
**3**	−305.20	1.85	−35.8
**4**	−295.2	28.2	−42.5
**5**	−303.90	1.58	1.4
**6**	−313.80	125.5	−46.2

**Table 2:** Results of the dark cytotoxicity study.

No.	Cell Cultures									
	S-37		PC-3		MCF-7		A-549		HeLa	
	Incubation Time after Drug Administration, Hours									
	24	48	24	48	24	48	24	48	24	48
	IC_50_, mmol/ml									
**2**	33.8 ± 1.4	5.9 ± 0.07	32.2 ± 1.3	10.3 ± 0.4	46.5 ± 0.7	26.9 ± 0.3	34.1 ± 1.1	9.5 ± 0.5	22.26 ± 0.3	8.0 ± 0.3
**3**	15.8 ± 3.1	12.8 ± 1.2	15.5 ± 0.9	5.5 ± 0.6	20.6 ± 0.3	16.5 ± 0.5	18.2 ± 1.1	8.5 ± 1.0	14.87 ± 0.4	16.8 ± 0.3
**4**	3.3 ± 0.3	3.3 ± 0.6	2.3 ± 0.7	1.0 ± 0.1	10.8 ± 0.2	3.9 ± 0.5	6.2 ± 0.9	2.8 ± 0.8	8.02 ± 0.8	10.0 ± 0.8
**5**	5.7 ± 0.9	2.7 ± 0.2	1.9 ± 0.5	1.1 ± 0.7	9.7 ± 0.4	6.9 ± 0.4	9.8 ± 0.5	3.0 ± 0.3	7.76 ± 0.2	8.6 ± 0.3
**6**	5.2 ± 1.5	3.1 ± 1.1	0.9 ± 0.4	0.6 ± 0.2	6.8 ± 0.1	3.1 ± 0.2	7.7 ± 1.1	2.2 ± 0.2	3.31 ± 0.1	3.0 ± 0.2

**Table 3:** Results of the photoinduced cytotoxicity study.

No.	Cell Lines									
	S-37		PC-3		MCF-7		A-549		HeLa	
	Incubation Time after Light Exposure, Hours									
	24	48	24	48	24	48	24	48	24	48
	IC_50_, mmol/ml									
**4**	0.21 ± 0.04	0.17 ± 0.01	0.14 ± 0.01	0.09 ± 0.04	0.19 ± 0.04	0.14 ± 0.04	0.23 ± 0.04	0.16 ± 0.01	0.66 ± 0.04	0.62 ± 0.05
**5**	0.23 ± 0.05	0.13 ± 0.04	0.08 ± 0.03	0.07 ± 0.05	0.22 ± 0.08	0.12 ± 0.05	0.18 ± 0.06	0.11 ± 0.02	0.47 ± 0.03	0.41 ± 0.01
**6**	0.2 ± 0.01	0.06 ± 0.02	0.08 ± 0	0.06 ± 0.04	0.1 ± 0.06	0.05 ± 0.01	0.13 ± 0.04	0.09 ± 0.03	0.32 ± 0.02	0.26 ± 0.02

## Data Availability

The data presented in this study are available from the authors.
